# Did You Know That

**Published:** 1916-12

**Authors:** 


### DID YOU KNOW THAT

It is dangerous to put anything into the mouth except food and drink?

---

Sanitary instruction is more important than sanitary legislation?

---

The United States Public Health Service issues free bulletins on tuberculosis?

---

The continuous liberal use of alcoholic beverages lowers efficiency and menaces longevity?

---

Moderate exercise in the open air prolongs life?

---

"Mouth breathing" makes children stupid?

---

Smallpox is wholly preventable?

